# Determining the gaps between Cochrane reviews and trials of effectiveness of interventions for acute respiratory infections: an audit

**DOI:** 10.1186/s13643-017-0472-0

**Published:** 2017-04-13

**Authors:** Jasmin Alloo, Sanya Vallath, Chris Del Mar, Matt Carter, Sarah Thorning, Justin Clark

**Affiliations:** 1 School of Medicine, Bond University, Gold Coast, Australia; 2 Cochrane Acute Respiratory Infections Group, Gold Coast, Australia; 3 Centre for Research in Evidence-Based Medicine, Bond University, Gold Coast, Australia; 4 Library, Gold Coast University Hospital, Gold Coast, Australia

**Keywords:** Cochrane reviews, Research gaps, Research prioritisation

## Abstract

**Background:**

Cochrane primarily aims to systematically review trials of effectiveness that are important to inform clinical decisions. Editorial groups support authors to achieve high-quality reviews and prioritise review proposals in their clinical domain that are submitted or elicited. Prioritising proposals requires two approaches, identifying (1) clinical practises for which the evidence of effectiveness is uncertain and (2) interventions in which there are trials of effectiveness (especially randomised controlled trials (RCTs)) not systematically reviewed. This study addresses this second approach for the Cochrane Acute Respiratory Infections Group (CARIG) in order to identify RCTs of acute respiratory infections that have not been systematically reviewed.

**Methods:**

We exported, on the 9th of September 2014, and then compared the group’s trials register of RCTs against a list of current Cochrane ARI (systematic) Reviews to identify gaps in topics (the same intervention and health condition) where completed trials have not been systematically reviewed. We assigned a principle intervention and health condition to each of 157 Cochrane reviews (CRs) and 5393 RCTs.

**Results:**

A majority of topics had been systematically reviewed; however, a substantial number (2174 or 41%) of RCTs were not included in any review. The topic that had been systematically reviewed the most was *antibiotic vs placebo for pneumonia* with 11 CRs and 205 RCTs. The topic that was the subject of most RCTs was *vaccination for influenza* with 525 RCTs and 6 CRs. Also, 6 CRs had no RCTs (‘empty reviews’).

**Conclusions:**

We identified many RCT topics that have not been systematically reviewed. They need to be addressed in a separate process to establish their priority to clinicians.

## Background

Systematic reviews (SRs) summarise and synthesise randomised controlled trials (RCTs), the best method for testing interventions, to produce high levels of evidence. Cochrane is an organisation committed to generating the highest level of evidence by systematically reviewing the medical literature [1]. It comprises 53 editorial groups. The Cochrane Acute Respiratory Infections Group (CARIG) focuses on reviewing and summarising the evidence of treatments for acute respiratory infections (ARIs) [2]. ARIs carry a large burden of disease [3].

Cochrane reviews are among the most rigorous forms of systematic review [4] and, accordingly, require the greatest editorial support provided by the Cochrane ARI Group. They typically take 6 months to 2 years to complete [5, 6]. Not all potential titles submitted can be supported, and so, a priority-setting process is necessary. [7–10]. As part of the CARIG’s priority-setting process, we resolved to determine interventions and health conditions for which there are RCTs not systematically reviewed by Cochrane.

## Methods

### Study selection and categorisation

We exported Cochrane reviews specific to the CARIG by interrogating Cochrane’s management software (‘Archie’), equivalent to searching the Cochrane Database of Systematic Reviews (CDSR) Issue 9 of 12, September 2014. We also exported a list of ARI-specific RCTs from the CARIG trials register (the date range of trials was 1930 to 2014). Both exports were done on the 9th of September 2014. Both lists were imported via a reference manager (EndNote) into a spreadsheet where two authors (JA and SV) independently examined the titles (and if necessary, abstracts) to classify the main interventions and health conditions (together forming a ‘topic’). Disagreements were settled by consensus or resolved by a third author (CDM).

We sorted CRs and RCTs by topic (the same intervention and health condition) and then matched CRs and RCTs with the same topic (or paired intervention and health condition). This process enabled us to identify the CRs and RCTS where a match was made on the same topic (intervention and health condition matched) and where there were existing RCTs on particular topics (intervention and health condition) but no CRs. We also identified where there were CRs on particular topics but no RCTs (empty reviews).

### Studies that did not consistently cover a single disease or intervention

For each CR or RCT, the intervention was classified followed by the health condition. Where more than one health condition and/or intervention was represented in a single CR or RCT, all health conditions and interventions were classified.

### Resolving categorisation discrepancies

Once all studies had been assigned an intervention and health condition, the categories were checked for consistency. Where appropriate, categories were merged together (e.g. the physiotherapy and exercise categories were merged into a single category called ‘physiotherapy/exercise’). Medical and common terms (e.g. ‘pharyngitis’ and ‘sore throat’) were also combined into a single category.

## Results

Out of 162 Cochrane reviews screened, 5 were excluded as they were either withdrawn or out of date, leaving 157 for inclusion. Out of 5393 RCT titles screened, 108 were excluded due to not addressing an ARI, had no intervention or were not an RCT, leaving 5285. Of these 409, required reading the abstract and, or, full text.

We initially listed 54 Cochrane review intervention categories, which we merged into 45, and 35 health condition categories were merged into 27. Similarly, 377 RCT intervention categories were merged to 182, and 168 health condition categories to 101.

The most common topics systematically reviewed by the CARIG were *antibiotics for pneumonia* (11 CRs, 6.4% of the total); *vaccination for influenza* (*n* = 6, 3.5%); *vaccination for pertussis* (*n* = 4, 2.3%); *antiviral drugs for influenza* (*n* = 4, 2.3%); *antibiotics for otitis media* (*n* = 4, 2.3%); and *antibiotics for sore throat* (*n* = 4, 2.3%) (Table 1). The most common interventions reviewed by the CARIG is *antibiotic vs placebo* (5 out of the top 10 most common topics). The topics which had been the focus of the most RCTS were *vaccinations for influenza* (525, 7.7%); *vaccination for pertussis* (303, 4.4%); and *antibiotic vs antibiotic for pneumonia* (269, 4%) (Table 2). The most commonly occurring intervention in the ARI trials register was *vaccination* (6 out of the top 10 most common topics). However, *antibiotic vs antibiotic* in general was the least common intervention Cochrane reviewed (only 1, 0.6%).

**Table 1:** Number of CRs and RCTs ranked by number of CRs

Intervention	Health condition	No. of RCTs	No. of CRs
Antibiotic vs placebo	Pneumonia	205	11
Vaccination	Influenza	525	6
Vaccination	Pertussis	303	4
Antiviral	Influenza	147	4
Antibiotic vs placebo	Otitis media	114	4
Antibiotic vs placebo	Pharyngitis/sore throat	90	4
Antibiotic vs placebo	ARI non-specific	129	3
CAM	Common cold	44	3
Antibiotic vs placebo	Meningitis	36	3
Vaccination	Diphtheria	246	2
Vaccination	Tetanus	236	2
Vaccination	Measles	161	2
Vaccination	Pneumococcus	143	2
Antibiotic vs placebo	Bronchitis, acute	60	2
Vaccination	Hepatitis	58	2
CAM	ARI non-specific	49	2
Antiviral	Herpes zoster	43	2
Antibiotic vs placebo	Bronchiolitis	42	2
Antihistamine	Common cold	41	2
Corticosteroid	Meningitis	39	2
Antitussive/decongestant/expectorant	Cough	36	2
Vaccination	Otitis media	35	2
Vaccination	Herpes zoster	30	2
Antibiotic vs placebo	Common cold	21	2
CAM	Influenza	18	2

**Table 2:** Number of CRs and RCTs ranked by number of RCTs

Intervention	Health condition	No. of RCTs	No. of CRs
Vaccination	Influenza	525	6
Vaccination	Pertussis	303	4
Antibiotic vs antibiotic	Pneumonia	269	0
Vaccination	Diphtheria	246	2
Vaccination	Tetanus	236	2
Antibiotic vs placebo	Pneumonia	205	11
Vaccination	Croup	188	1
Vaccination	Measles	161	2
Antiviral	Influenza	147	4
Antibiotic vs antibiotic	Bronchitis, acute	146	0
Vaccination	Pneumococcus	143	2
Antibiotic vs antibiotic	Pharyngitis/sore throat	140	0
Antibiotic vs antibiotic	Otitis media	135	0
Antibiotic vs placebo	ARI non-specific	129	3
Vaccination	Meningitis	116	1
Antibiotic vs placebo	Otitis media	114	4
Antibiotic vs antibiotic	ARI non-specific	106	0
Antibiotic vs placebo	Pharyngitis/sore throat	90	4
Vaccination	Mumps	86	1
Antibiotic vs antibiotic	Sinusitis	82	0
Vaccination	Rubella	80	1
Vaccination	Polio	67	0
Immunotherapy	ARI non-specific	65	1
Antibiotic vs placebo	Bronchitis, acute	60	2
Antibiotic vs placebo	Sinusitis	60	1

There were many RCTs with no corresponding CRs (2174 or 41%) (Table 3). Most used the intervention of *antibiotics*, which accounts for 878 RCTs (12.8%). Similarly, there were (only) 6 CRs which reviewed no RCTs (that is they were ‘empty reviews’) (Table 4).

**Table 3:** RCTs with no CR ranked by number of RCTs

Intervention	Health condition	No. of RCTs	No. of CRs
Antibiotic vs antibiotic	Pneumonia	269	0
Antibiotic vs antibiotic	Bronchitis, acute	146	0
Antibiotic vs antibiotic	Pharyngitis/sore throat	140	0
Antibiotic vs antibiotic	Otitis media	135	0
Antibiotic vs antibiotic	ARI non-specific	106	0
Antibiotic vs antibiotic	Sinusitis	82	0
Vaccination	Polio	67	0
Antibiotic vs antibiotic	Bronchiolitis	53	0
NSAID	ARI non-specific	36	0
Immunotherapy	Common cold	34	0
NSAID	Pharyngitis/sore throat	31	0
Antiviral	Common cold	27	0
Vaccination	Herpes simplex	25	0
Vitamin A	ARI non-specific	23	0
Antitussive/decongestant	Otitis media	23	0
Vitamins and supplements	ARI non-specific	19	0
Antibiotic vs placebo	Staphylococcus	19	0
Vaccination	ARI non-specific	18	0
CAM	Bronchiolitis	17	0
Vaccination reminder	Influenza	17	0
Humidification/steam	Pneumonia	16	0
Antiviral	Respiratory syncytial virus	16	0
Vaccination	Respiratory syncytial virus	15	0
Antibiotic vs antibiotic	Staphylococcus	15	0
Antibiotic vs antibiotic	Streptococcus	14	0

**Table 4:** CRs with no RCTs (empty reviews)

Intervention	Health condition	No. of RCTs	No. of CRs
Zinc	Otitis media	0	1
Acupuncture	Mumps	0	1
CAM	Bronchitis, acute	0	1
CAM	Mumps	0	1
Fluid therapy	ARI non-specific	0	1
Nasal irrigation	ARI non-specific	0	1

**Fig. 1:**
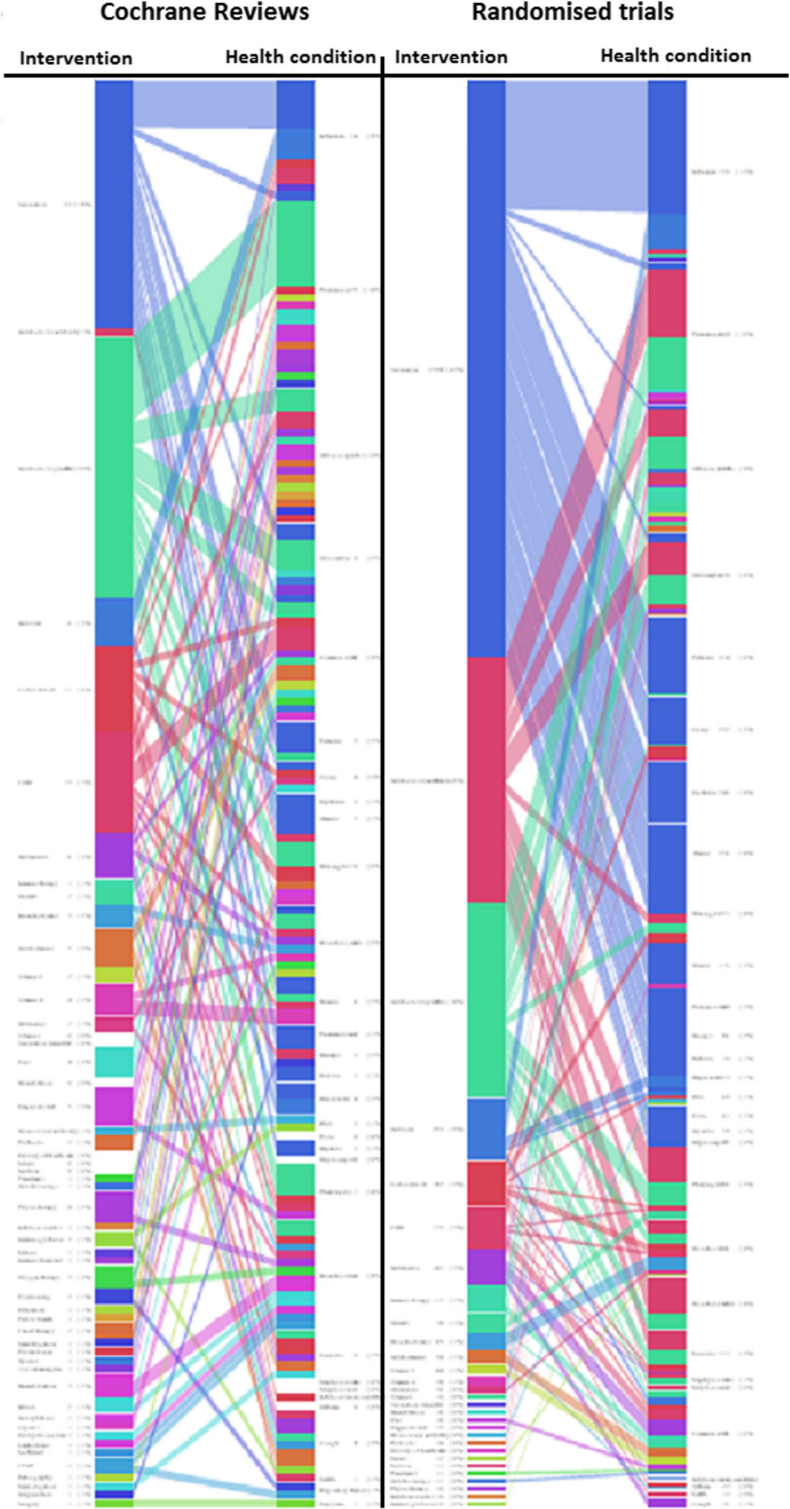
Graphical representation of CRs mapped to RCTs overview. *Colours* are used to identify the same interventions, for instance *blue*, as seen here, represents *vaccination*. In the live version, users can select the topic of interest by passing the mouse over it, which will expand the number of RCTs and CRs associated with that topic. The figure shows a static sub-section of the graph at http://crebp.github.io/CREBP-Disease-Treatment/

**Fig. 2:**
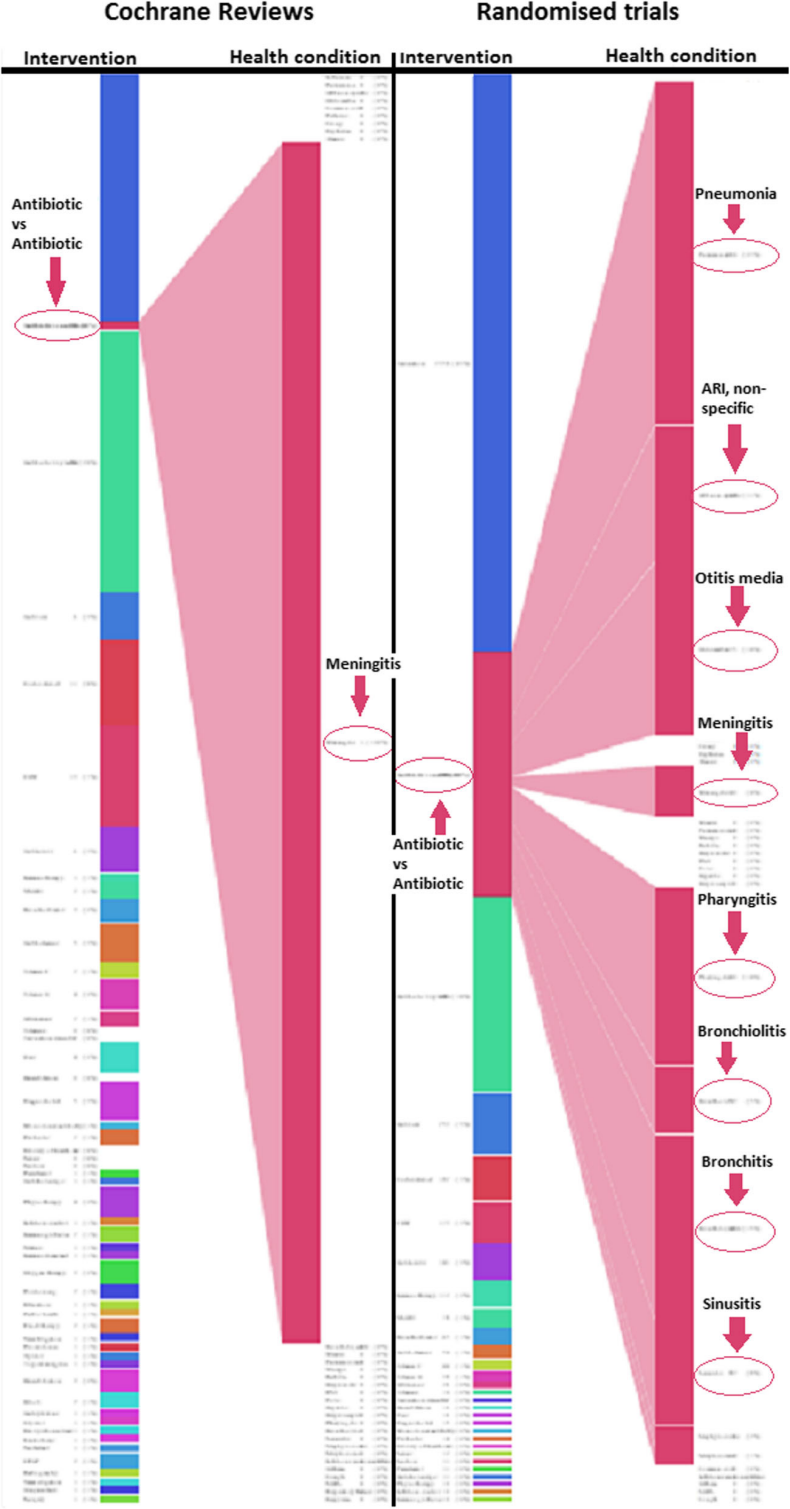
Graphical representation of Cochrane review mapped to RCTs (*antibiotic vs antibiotic*). This static screenshot shows the effects of a reader’s mouse over an intervention which is covered by a Cochrane review (here, *antibiotic vs antibiotic*): the right side of the figure expands to show the diseases for which *antibiotic vs antibiotic* has been studied

**Fig. 3:**
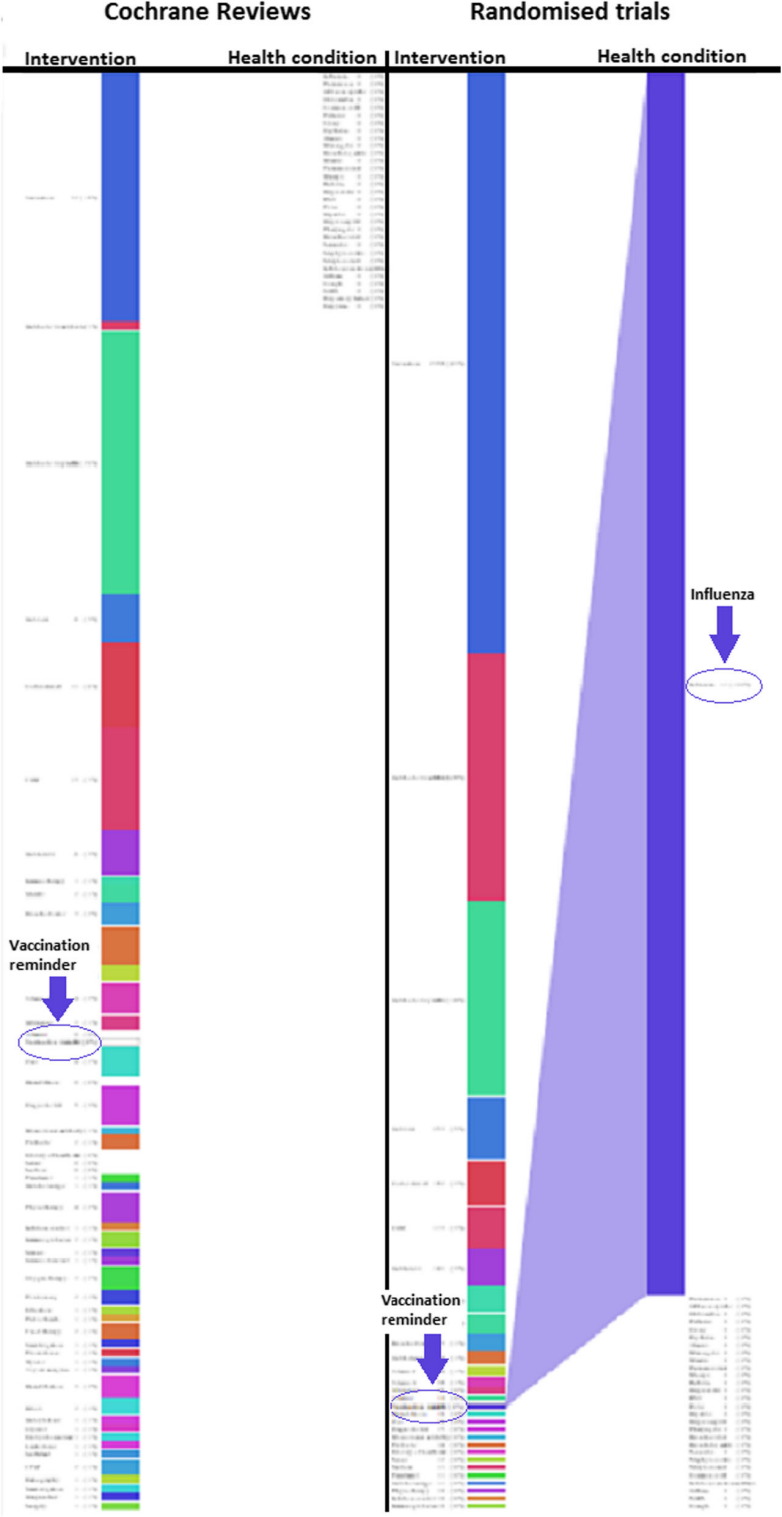
Graphical representation of Cochrane reviews mapped to RCTs (no CR, vaccination reminder). In this example, there is an intervention (*vaccination reminders*) with RCTs but no corresponding Cochrane review (a ‘review gap’). The RCT section has expanded to show the topics with available evidence that could be incorporated into a Cochrane review. The Cochrane review section is empty

We devised a novel method of representing the extensive relationship between CRs and RCTs, which conveys the information in Tables 1, 2 and 3 that allows the user to interact with the data (Figs. 1, 2 and 3). This is an interactive online graph available from our website [11].

## Discussion

We found many topics which have been trialled but not reviewed, consistent with the previous findings [12]. These data should allow the CARIG to identify clinical questions in need of review.

The topic with the most Cochrane reviews was *antibiotics for pneumonia*. Pneumonia makes an important contribution to the burden of disease worldwide, especially in the developing world [3], and so, this over-representation seems appropriate. The interventions trialled with least representation with CRs, *antibiotic vs antibiotic*, are often driven by pharmaceutical companies (interested in demonstrating that a new member of an existing antibiotic class has equivalent efficacy), something perhaps less interesting to clinicians.

Strengths of our methods were the exploitation of the set of trials already collected by Cochrane and the collection of CRs and our matching methods and online visualisation techniques. Weaknesses include the limitation to CRs (there are undoubtedly other systematic reviews outside Cochrane), the potentially arbitrary over-simplification of the topics to one or two interventions for each health condition category and our limitation to treatment questions (Cochrane has a minority of diagnostic reviews as well)—nor did we account for ‘stabilised reviews’ (those in which the intervention is no longer current, e.g. *amantadine and rimantadine for influenza* [13] or where there is sufficient evidence to settle for a clinical question), e.g. *vaccination to prevent polio* [14].

## Conclusions

These data will inform our forthcoming priority-setting exercise during which they will be presented to stakeholders (health consumers and clinicians) to allow judgement to be made about which topics should be given higher priority.

## Abbreviations

ARI: Acute respiratory infections
CARIG: Cochrane Acute Respiratory Infections Group
CDSR: Cochrane Database of Systematic Reviews
CR: Cochrane review
RCT: Randomised controlled trial
SR: Systematic review

## References

[1] Cochrane: about us. http://www.cochrane.org/about-us. Accessed 22 Dec 2016.

[2] Cochrane Acute Respiratory Infections: about us. http://ari.cochrane.org/welcome. Accessed 22 Dec 2016.

[3] Ferkol T, Schraufnagel D (2014). The global burden of respiratory disease. Ann Am Thorac Soc.

[4] Useem J, Brennan A, LaValley M, Vickery M, Ameli O, Reinen N, Gill CJ (2015). Systematic differences between Cochrane and non-Cochrane meta-analyses on the same topic: a matched pair analysis. PLoS One.

[5] Ganann R, Ciliska D, Thomas H (2010). Expediting systematic reviews: methods and implications of rapid reviews. Implement Sci.

[6] Tricco AC, Antony J, Zarin W, Strifler L, Ghassemi M, Ivory J, Perrier L, Hutton B, Moher D, Straus SE (2015). A scoping review of rapid review methods. BMC Med.

[7] Cochrane Methods priority setting: background & history. http://methods.cochrane.org/prioritysetting/background-history. Accessed 18 Dec 2016.

[8] Synnot A (2016). Stakeholder priorities for research in health communication and participation: findings from the Cochrane consumers and communication priority setting project.

[9] Cochrane tobacco addiction: priority setting workshop http://tobacco.cochrane.org/priority-setting-workshop. Accessed 16 Dec 2016.

[10] Welsh E, Stovold E, Karner C, Cates C (2015). Cochrane Airways Group reviews were prioritized for updating using a pragmatic approach. J Clin Epidemiol.

[11] Cochrane ARI Group. Topics covered (systematic reviews) vs. not-covered (no systematic reviews) by the Cochrane ARI Group http://crebp.github.io/CREBP-Disease-Treatment/. Accessed 15 Jan 2017.

[12] Glasziou P, Haynes B (2005). The paths from research to improved health outcomes. ACP J Club.

[13] Jefferson T, Demicheli V, Di Pietrantonj C, Rivetti D. Amantadine and rimantadine for influenza A in adults. Cochrane Database Syst Rev. 2006 19;(2):CD001169. 10.1002/14651858.CD001169.pub3 PMC7068158 16625539

[14] CDC. Polio vaccination. https://www.cdc.gov/vaccines/vpd/polio/index.html. Accessed 19 Dec 2016.

[15] Alloo J, Vallath S, Del Mar C, Carter M, Thorning S, Clark J. Determining the gaps between Cochrane reviews and trials of effectiveness of interventions for acute respiratory infections: an audit (Data set). In: Gold Coast: Bond University; 2016. 10.1186/s13643-017-0472-0 PMC5390471 28407780

